# Metabarcoding reveals a high diversity of woody host-associated *Phytophthora* spp. in soils at public gardens and amenity woodlands in Britain

**DOI:** 10.7717/peerj.6931

**Published:** 2019-05-16

**Authors:** Carolyn E. Riddell, Debbie Frederickson-Matika, April C. Armstrong, Matt Elliot, Jack Forster, Pete E. Hedley, Jenny Morris, Peter Thorpe, David EL Cooke, Leighton Pritchard, Paul M. Sharp, Sarah Green

**Affiliations:** 1 Forest Research, Roslin, Midlothian, UK; 2 The Woodland Trust, Edinburgh, UK; 3 Forest Research, Farnham, Surrey, UK; 4 James Hutton Institute, Dundee, UK; 5 School of Medicine, University of St. Andrews, St Andrews, UK; 6 Institute of Evolutionary Biology, University of Edinburgh, Edinburgh, UK

**Keywords:** *Phytophthora*, Metabarcoding, ITS1 barcode, Illumina sequencing, Soil, Species diversity

## Abstract

Forests and woodlands worldwide are being severely impacted by invasive *Phytophthora* species, with initial outbreaks in some cases occurring on host trees located in public parks and gardens. These highly disturbed sites with diverse planting practices may indeed act as harbours for invasive *Phytophthora* pathogens which are particularly well adapted to surviving in soil. High throughput Illumina sequencing was used to analyse *Phytophthora* species diversity in soil samples collected from 14 public garden/amenity woodland sites in northern Britain. Bioinformatic analyses revealed some limitations to using internal transcribed spacer as the barcode region; namely reporting of false positives and ambiguous species matches. Taking this into account, 35 distinct sequences were amplified across the sites, corresponding to 23 known *Phytophthora* species as well as twelve oomycete sequences with no match to any known *Phytophthora* species. *Phytophthora pseudosyringae* and *P. austrocedri*, both of which cause serious damage to trees and are regarded as fairly recent introductions to Britain, were the two most abundant *Phytophthora* species detected. There was no evidence that any of the detected *Phytophthora* species were more associated with any one type of host, healthy or otherwise. This study has demonstrated the ubiquity and diversity of *Phytophthora* species endemic in highly managed, extensively planted soil environments in Britain. Suggested improvements to the methodology and the practical implications of the findings in terms of mitigating *Phytophthora* spread and impact are discussed.

## Introduction

Invasive pests and pathogens are of increasing concern globally, largely due to human-mediated intercontinental spread via trade and other pathways (Hulbert et al., 2017). Often, the first documented outbreaks of invasive organisms are recorded in urban environments, and this is frequently observed in the case of pathogens of woody hosts where public parks, gardens and arboreta can act as reservoirs of invasive populations which go on to invade local ecosystems, known as the ‘bridgehead effect’ (Paap, Burgess & Wingfield, 2017a). *Phytophthora*, a diverse genus of filamentous oomycete plant pathogens, has provided some of the most notorious examples of invasive and highly destructive plant disease epidemics worldwide. These include the potato late blight pathogen *P. infestans* (Goss et al., 2014) and the two forest pathogens, *P. cinnamomi* (Dobrowolski et al., 2003) and *P. ramorum* (Goss, Carbone & Grünwald, 2009). Although fungus-like, *Phytophthora* species are more closely related to brown algae and are taxonomically positioned within the Kingdom Stramenopila, producing motile, free-swimming zoospores which are formed in spore sacs known as sporangia (Erwin & Ribeiro, 1996). *Phytophthora* species also produce thick-walled resting spores that are very resilient to degradation and able to survive in plant residues and soils over years or even decades once the pathogen has become established on a site (Ristaino & Gumpertz, 2000). Currently, approximately 180 species of *Phytophthora* have been provisionally named worldwide, with new cryptic species being described at an increasing rate as a result of global surveys for *Phytophthora* in many environments. Recent multigene phylogenies have divided *Phytophthora* into ten (Yang, Tyler & Hong, 2017) or 12 (Jung et al., 2017a) clades. The number of clades will no doubt expand as the true global diversity of this genus of plant pathogens is revealed, with some estimates suggesting that the total number of species could be close to 500 (Brasier, 2008).

Since much of the *Phytophthora* life cycle takes place in roots, soil or water, the diseases they cause may be difficult to diagnose and control (Erwin & Ribeiro, 1996). Worldwide, the impact of *Phytophthora* on trees in both natural and managed ecosystems has increased greatly in recent years, due in part to the inadvertent movement of these pathogens in traded plants (Jung et al., 2016). In Britain, for example, five new species of pathogenic *Phytophthora* (*P. ramorum*, *P. kernoviae*, *P. lateralis*, *P. austrocedri* and *P. pseudosyringae*) have been reported since 2003, all causing serious damage to trees across a range of different environments and resulting in significant economic and ecological losses (Green & Webber, 2012). In almost all of these cases, imported planting material has been either confirmed or strongly implicated as the most likely route of introduction. Three of these pathogens (*P. lateralis*, *P. pseudosyringae* and *P. austrocedri*) were discovered as a result of disease outbreaks on trees at public parks and gardens, all being highly disturbed sites with extensive planting histories. A recent analysis of documented records by Cooke (2015) found forty-two *Phytophthora* species recorded as present in Britain with about 13 of these species known to be associated with woody hosts and the rest associated with herbaceous hosts or water courses. Future invasions of *Phytophthora* from other global sources are likely because of their ability to survive in soil unseen, the difficulties of effective disease surveillance given the high annual volumes of imported plants and associated soil, and the limitations of current molecular and biological detection techniques, particularly when a previously undescribed species is present.

High throughput metabarcode sequencing is a rapidly advancing technology which has the potential to detect all species of a target genus present within an environmental sample, including species as yet undescribed (Mendoza, Sicheritz-Pontén & Gilbert, 2015). Previous studies have demonstrated the power of metabarcoding for analyses of *Phytophthora* diversity in soil using a method based on 454 pyrosequencing. These include Vannini et al. (2013) who detected 15 *Phytophthora* species in soils sampled from two *Castanea sativa* forests in Italy compared with only four species detected from the same soil samples using a traditional baiting method, Català, Pérez-Sierra & Abad-Campos (2015) who identified 13 different *Phytophthora* species in soil samples collected from two forest sites in Spain, and Burgess et al. (2017, 2018) who analysed the distribution and diversity of *Phytophthora* in 640 soil samples collected across several Australian provinces. The latter authors found 68 distinct *Phytophthora* phylotypes comprising 21 potentially novel taxa and 25 species that were new records either to Australia or to natural ecosystems in the country. Most recently, a study comparing the community composition and distribution of *Phytophthora* species in adjacent native and non-native forests in South Africa recorded 32 *Phytophthora* taxa using metabarcoding compared with five taxa using baiting (Bose et al., 2018), further demonstrating the superiority of metabarcoding over baiting in environmental analyses of *Phytophthora* species diversity.

Based on such findings it is hypothesised that the diversity of *Phytophthora* species present in highly managed and publicly accessible wooded garden sites in Britain is greater than current evidence suggests, and will include invasive species now causing damage to trees in wider commercial forestry and natural woodland settings as well as species not previously recorded in the country. Metabarcoding, applied to such sites, may also be a potentially useful tool for routine surveillance aimed at early detection of invasive pathogens soon after their introduction. Here, we apply Illumina sequencing technology, which provides more reads of a higher quality and lower error rates than 454 pyrosequencing, to test the applicability of the metabarcoding method for analyses of *Phytophthora* diversity in soils, targeting what we consider to be high-risk sites; namely public parks, botanic gardens and other woodland sites with histories of plant importation and documented *Phytophthora* outbreaks. We discuss the biological significance of our findings and some observed limitations to the metabarcoding method. The practical implications in terms of disease surveillance and other strategies to mitigate further *Phytophthora* impact and spread in highly managed, publicly accessible wooded environments are also discussed.

## Materials and Methods

### Soil sampling and DNA extraction

Fourteen sites in northern Britain were sampled between October 2014 and September 2015. These included eleven public gardens/arboreta, two public cemeteries and one privately owned upland juniper woodland with no public access. With the exception of one of the public gardens, all sites had previously confirmed *Phytophthora* outbreaks. The geographical coordinates (not shown here) and approximate altitude (Table S1) were determined for each site from a roughly central location. For ten of the sites the underlying soil type was determined using the Soil Information for Scottish Soils web interface http://sifss.hutton.ac.uk/ (Table S1). For the remaining four sites (sites 2, 4, 5 and 14) the underlying soil type could not be determined due to their location in urban areas (Table S1). At each site, soil samples were collected from around ten trees/shrubs either showing visible symptoms typical of *Phytophthora* infection such as stem bleeding and foliage browning/bronzing, or which were outwardly healthy but known to be potential *Phytophthora* hosts. Soil was also sampled from around stumps of trees/shrubs recently felled due to *Phytophthora* disease. Thus the locations of sampling at each site varied according to the above criteria, with sampling points distributed over as wide an area on each site as possible. For each sampling point the geographical coordinates were recorded as well as the genus/species of tree/shrub present and its condition, either; i) dead, stump ii) dead, standing, iii) live, but showing symptoms typical of *Phytophthora* infection and iv) live, outwardly healthy. Table S1 shows the number of healthy vs diseased trees/shrubs, and their broad taxonomic grouping, that formed each soil sampling point across the 14 sites.

Each individual soil sample (approximately 300–400 g) was comprised of eight pooled soil cores of 2 cm width × 30 cm depth collected using a soil auger whereby two cores were collected at four points around each individual tree/shrub/stump within one m horizontal distance from the root collar. The eight subsamples were pooled and homogenised by hand in a single grip-seal™ polythene bag and frozen within 12 h of collection. Each soil sample was oven dried at ∼60 °C in aluminium trays for 1–3 days (depending on soil wetness), stirred thoroughly once dry and DNA extracted from three 250 mg subsamples using the PowerSoil® DNA isolation kit (MoBio Laboratories Inc., Carlsbad, CA, USA). A robotic workstation for DNA extraction based on magnetic-particle purification (Kingfisher™ mL Magnetic Particle Processor, Thermo Scientific, Loughborough, UK) was used for the DNA extraction process. Post-DNA extraction clean-up was carried out using either the Jet-Quick™ DNA purification kit (Genomed GmbH, Löhne, Germany) or DNA Clean & Concentrator™ (Zymo Research, Irvine, CA, USA) according to the manufacturer’s instructions.

### Development of positive controls for all Illumina sequencing runs

For the first sequencing run a control genomic DNA mix was set up containing the following 15 *Phytophthora* species; *P. austrocedri*, *P. cactorum*, *P. cambivora*, *P. chlamydospora*, *P. cinnamomi*, *P. gonapodyides*, *P. ilicis*, *P. kernoviae*, *P. lateralis*, (EU2 lineage), *P. obscura*, *P. plurivora*, *P. pseudosyringae*, *P. ramorum*, *P. syringae* and *P. boehmeriae* (all but the last known to be present in the UK). For the second sequencing run a control genomic DNA mix was set up containing the following ten *Phytophthora* species; *P. cactorum*, *P. foliorum*, *P. obscura*, *P. plurivora*, *P. rubi*, *P. siskiyouensis* (all known to be present in the UK), and *P. boehmeriae*, *P. castaneae*, *P. capsici* and *P. fallax* (not known to be present in the UK). Species were included in the control mixes based on being present in Britain and expected to be detected in soil samples, as well as species not present in Britain and therefore not expected to be detected. Species were also chosen to span as wide a range of clades as possible from within these two criteria, with seven clades represented in both mixes. All cultures originated from single hyphal-tip colonies. Genomic DNA was extracted from mycelium harvested from *Phytophthora* cultures on V8 agar using the Nucleospin Plant Prep II kit (Machery-Nagel, Düren, Germany) according to the manufacturer’s instructions. A SYBR green quantitative real-time PCR assay was carried out on an ABI Prism 7300 Real Time PCR System (Applied Biosystems, Foster City, CA, USA) with the primers 18Ph2F and 5.8S-1R (Scibetta et al., 2012) using DNA of each *Phytophthora* species diluted 5, 10, 25, 50, 100 and 500×. Cycle threshold (Ct) values at each concentration were determined and DNAs from each species corresponding to Ct values between 22 and 30 were pooled for the DNA control mix; thus species were not expected to be present in equal abundance.

### Amplicon PCR

A ∼ 250 bp region of the ribosomal RNA (rRNA) internal transcribed spacer (ITS1) was amplified from each DNA sample using nested PCR with primer pairs 18Ph2F and 5.8S-1R in the first round and ITS6 and 5.8S-1R in the second round according to the protocol of Scibetta et al. (2012), except that proof-reading enzyme KAPA HiFi HotStart ReadyMix (KAPA Biosystems, Wilmington, MA, USA) was used for the PCR reaction to minimise errors during PCR. Second round primers were amended with overhang adapters to ensure compatibility with the Illumina index and sequencing adapters. These were: forward overhang; 5′ TCGTCGGCAGCGTCAGATGTGTATAAGAGACAG‐ [ITS6] and reverse overhang; 5′ GTCTCGTGGGCTCGGAGATGTGTATAAGAGACAG‐ [5.8S-1R] (Illumina, 2013). For each DNA sample, PCR was carried out in triplicate and all *Phytophthora*-positive PCR replicates were pooled for downstream processing.

### Illumina sequencing library preparation and sequencing

Samples were prepared for sequencing following the protocols described for 16S Metagenomic Sequencing Library Preparation (Illumina, 2013). In brief, this involved clean-up of amplicon PCR using Agencourt® AMpure® XP beads (Agencourt Bioscience, Beverly, MA, USA) followed by index PCR in KAPA HiFi HotStart ReadyMix (KAPA Biosystems, Wilmington, MA, USA) to attach dual indices and Illumina sequencing adapters to each sample using the Nextera XT Index Kit (Epicentre, Madison, WI, USA). This step ensured that each sample could be uniquely identified during the sequencing run. A second PCR clean-up (as above) was then carried out and DNA of each sample visualised on an Agilent 2200 TapeStation (Santa Clara, CA, USA) and quantified using a Qubit® fluorimeter (Life Technologies, Paisley, UK). For each sequencing run, 96 samples were pooled for paired-end (2 × 250 bp) sequencing on a single flow cell of an Illumina MiSeq sequencer using the MiSeq v. 2 500 bp Nano kit (Illumina, San Diego, CA, USA) at the James Hutton Institute, Dundee, UK. Following quality control and de-multiplexing, FASTQ files containing reads for each sample were exported for bioinformatics analysis.

### Bioinformatics analysis

Sequence data from all 14 sites were processed in an attempt to assign them to clusters of highly similar sequences, referred to hereafter as operational taxonomic units (OTUs), and assigned species identity using the bioinformatics software ‘metapy’ (https://github.com/peterthorpe5/public_scripts/tree/master/metapy) (github commit: 6fd1864). The version of metapy used in this study performed read quality trimming using Trimmomatic (version 0.36) (Bolger, Lohse & Usadel, 2014). Reads were then assembled using PEAR (version 0.9.10) (Zhang et al., 2013). Metapy subsequently ran two sequence analysis tools which performed independently of each other. These were Swarm (version 1.2.19) (Mahé et al., 2014) and Bowtie (version 2.2.5) (Langmead, 2010). Swarm calculates sequence differences between pairs of reads to delineate OTUs using *k*-mer comparisons and a global pairwise alignment algorithm. Clustering results are refined based on sequence abundance and OTU structure (Mahé et al., 2014). For the analyses reported here the Swarm parameters set to delineate OTUs were ‘t -1 d -1’ and corresponded to approximately one to two mismatches across the sequence. Bowtie is a read-mapping tool and metapy filtered the resulting output for perfect matches. Species identification was based on a version of the custom curated *Phytophthora* reference database developed by Català, Pérez-Sierra & Abad-Campos (2015) which was updated to include more recently described *Phytophthora* species plus additional ITS1 sequences for species where intra-species ITS1 variability has been documented. This database version was dated June 2017 (https://github.com/peterthorpe5/public_scripts/blob/master/metapy/data/Riddell_et_al_2019.fasta). Any sequences not assigned species identity from the reference database were compared to the GenBank nt database using BLASTN+ (Altschul et al., 1990) to find the nearest matching sequences. Sequences that did not match to the reference database but which were close to *Phytophthora* sequences in the GenBank nt database were subjected to phylogenetic analyses to show their position within their clade. Table S3 shows the GenBank accession numbers and isolate identifiers for sequences used in the phylogenetic analyses. Maximum likelihood (ML) trees were estimated from nucleotide sequences using PhyML 3.0 (Guindon et al., 2010), with the general time reversible (GTR) model of sequence change and gamma-distributed rate variation across sites (four categories); 1,000 bootstrap replicates were examined. Midpoint-rooted phylogenies were displayed using FigTree (http://tree.bio.ed.ac.uk/software/figtree/).

### Statistical analyses

To improve the balance of the data across sites and reduce the number of factors, host health status was defined as ‘healthy’ or ‘symptoms/stump/dead’ (initial analysis indicated no significant differences among symptomatic, stump or dead samples). Table S1 shows the counts of samples, broken down by the relevant site-level and within-site factors considered within the statistical analysis. The ordination of *Phytophthora* assemblages was conducted using both abundance data (i.e. number of reads) and absence/presence of *Phytophthora* species. *Phytophthora* species were excluded from the ordination analysis when they appeared in only a single sample, and samples were excluded where no *Phytophthora* species were detected. For abundance data, non-metric multidimensional scaling (NMDS) was conducted on the Bray–Curtis dissimilarity matrix, with centring and Wisconsin square-root transformations. For absence/presence data, a Jaccard distance matrix was calculated using the binary data set. In both cases, ordination was conducted using the metaMDS function in the vegan package version 2.5-2 in R (Oksanen et al., 2018), with three dimensions required to produce acceptable stress levels (stress = 0.153 and 0.126 respectively). For statistical analysis, permANOVA was conducted on matrices for both data sets using the adonis functions in the vegan package in R: for drivers of site-level differences, latitude, longitude, altitude and underlying soil type were used as potential factors and variables, with sites used as strata within the models. A separate analysis was subsequently conducted for the effects of host status and taxonomic group within site, with marginal effects calculated in each case.

Generalised linear mixed-effects models (GLiMMs) were also applied to the data set for all samples (*n* = 140), but restricted to *Phytophthora* species present in more than 10 samples, as inclusion of rarer species prevented valid model fit. Data were analysed as absence/presence (i.e. binary) data of each individual *Phytophthora* species, with binomial error structure and probit link function, with host status/*Phytophthora* species interaction, host taxonomic group and site-level factors/variables as fixed effects, and sample nested within site as random effects. Analysis of variance (Wald chi-square tests) was applied to the model to assess the significance of the main effects and interactions; non-significant effects were subsequently removed to determine the best-fit model. The best-fit model residuals were assessed using the DHARMa package version 0.2.0 in R (Hartig, 2018). Post hoc analysis (Tukey’s HSD) was used to determine significant differences within significant effects, with outputted marginal means, i.e. averaged over significant factors, representing the proportion of samples with *Phytophthora* species present.

### Baiting

Soils were additionally baited for *Phytophthora* from one of the botanic garden sites (site 12; Table S1). Soil samples were collected in June 2016 from around five of the trees/shrubs originally sampled in October 2014. These sampling points were chosen based on having yielded a diverse mix of *Phytophthora* sequences in the metabarcoding analysis. For each baited sample approximately 250 g of soil was obtained by pooling four soil cores collected within one horizontal metre of the tree/shrub stem base. Samples were stored at 4 °C and baiting carried out on the soil at two different states; i) after 1 week storage at 4 °C, and ii) air dried at room temperature (18–22 °C) after the first baiting test followed by 5 weeks storage at 4 °C. Soil was baited inside wounded apples of the variety ‘Granny Smith’, two apples per soil sample, with one apple incubated at room temperature and the other incubated at 16 °C. Soil baiting with plant material was also set up whereby each soil sample was placed in a plastic sandwich box which was filled to twice the depth of the soil with distilled water and left to settle for 24 h. Any floating soil material was then removed and intact leaves of the following species placed to float on the water in each sample box as follows; 2 × *Rhododendron ponticum* leaves, 2× *Quercus robur* leaves, 6× *Hebe* leaves, 3× *Hebe* cuttings, 1× *Juniperus communis* cutting and one leaf each of *Solanum lycopersicum*, *Capsicum annuum*, *Petunia* sp., *Dianthus* sp. and *F. sylvatica*. Controls were also set up in which each baiting test was replicated in the absence of soil.

Bait plants were examined every 2 days for lesion development. Tissue from lesion margins was plated onto Synthetic Mucor Agar of Elliot, Hendrie & Knights (1966) with the addition of antibiotics as described in Brasier et al. (2005) and incubated at 16 °C. Developing colonies were subcultured onto V8 juice agar (V8A; 2 g CaCO_3_, 200 ml V8 juice and 15 g agar in 800 ml distilled water), incubated at 16 °C and, once grown, three 1 cm^3^ plugs were cut from the edge of each colony and transferred to Petri plates containing fresh pond water pre-filtered through Whatman No. 1 filter paper (GE Healthcare, Little Chalfont, UK). The plates containing pond water were incubated at 16 °C for 3–4 days and checked for the presence of *Phytophthora*-like sporangia.

DNA was extracted from mycelium harvested from *Phytophthora*-like cultures on V8 agar using the Nucleospin Plant Prep II kit (Machery-Nagel, Düren, Germany) according to the manufacturer’s instructions. PCR was performed using one µl of either the *Phytophthora*-specific forward primers Ph2 (Ippolito, Schena & Nigro, 2002) or ITS6 (White et al., 1990), and the universal reverse primer ITS4 (White et al., 1990) at a concentration of 10 mM. Total reaction volume was 25 µl comprising 1.5 µl MgCl_2_ (at 0.45 mM), five µl of 5× buffer, 0.5 µl dNTPs (at 0.2 mM), 0.125 µl U Taq DNA polymerase (Bioline, London, UK), 17.5 µl molecular grade water and one µl of template DNA. Amplification was performed in a Veriti 96 well Thermalcycler (Applied Biosystems, Foster City, CA, USA) with initial denaturation at 95 °C for 5 min followed by 35 cycles of 94 °C for 30 s, 55 °C for 30 s, 72 °C for 1 min and a final extension of 72 °C for 7 min. PCR products were purified and sequenced in both directions with the BigDye version 3.1 Ready Reaction Kit on an ABI Prism 3730 capillary sequencer (Applied Biosystems, Foster City, CA, USA). Raw sequences were aligned and edited using Sequencher version 4.8 for Windows, and searched against published ITS sequences in the GenBank nt database using BLASTN+ (Altschul et al., 1990).

## Results

### Sequencing output and performance of clustering tools in the control reactions

The two sequencing runs, which included samples from the 14 sites and two control reactions, together generated >400,000 good quality sequences that could be considered for analysis. A total of 7,123 assembled sequences were generated from the control reaction comprising 15 *Phytophthora* species included in the first sequencing run. In the second sequencing run a total of 4,459 assembled sequences were generated from the control reaction comprising ten *Phytophthora* species. Using the metapy software, the two sequence analysis tools Bowtie and Swarm reported different numbers of true positive and false positive *Phytophthora* species for each control reaction, and different numbers of species not detected in each reaction (Table 1).

**Table 1:** Number of true positive and false positive *Phytophthora* species reported in two DNA control mixes by two sequence analysis tools in the metapy pipeline with the database used in this study.

Tool	True positive	False positive	Sensitivity^a^	Precision^b^	False discovery rate^c^	False negative rate^d^
	Control mix 1 (15 *Phytophthora* species)					
Bowtie	11	1	0.73	0.92	0.08	0.27
Swarm	14	4	0.93	0.78	0.22	0.07
	Control mix 2 (10 *Phytophthora* species)					
Bowtie	7	6	0.70	0.54	0.46	0.30
Swarm	9	10	0.90	0.47	0.53	0.10
	Overall performance over both control runs					
Bowtie	18	7	0.72	0.72	0.28	0.28
Swarm	23	14	0.92	0.62	0.38	0.08

**Notes:**

a Calculated as the number of true positives/(number of true positives + number of false negatives).

b Calculated as the number of true positives/(number of true positives + number of false positives).

c Calculated as the number of false positives/(number of true positives + number of false positives).

d Calculated as the number of false negatives/total number of species included in the sample.

Using the Bowtie tool, species included in the control mixes but not identified were *P. boehmeriae*, *P. gonapodyides*, *P. ilicis* and *P. syringae* in the first control mix and *P. boehmeriae*, *P. cactorum* and *P. siskiyouensis* in the second control mix. False positive species assigned to OTUs using Bowtie were *P. idaei* in the first control reaction and *P. glovera*, *P. agathacidica*, *P. sp. novaeguineae*, *P. citricola*, *P. fragariae* and *P. idaei* in the second control reaction (Table 2). The number of reads allocated to an OTU using Bowtie ranged from 258 to one in the first control reaction and 192 to one in the second control reaction (Table 2). For two of the OTUs the false positives were due to shared ITS1 sequences preventing discrimination of very closely related species. These were *P. glovera*, which has an identical ITS1 sequence to *P. capsici*, and *P. agathacidica* and *P. sp. novaeguineae* which have identical ITS1 sequences to *P. castanae*. Since Bowtie was used with settings that assign species to OTUs only when there are no mismatches across the alignment, the remaining false positive results were potentially ITS1 sequence variants within the genomes of individual strains included in the control mix. Excluding *P. boehmeriae*, which is discussed later, the false-negative results and overall low number of reads assigned to each OTU using Bowtie are probably due to differences between the reference sequences in the database and sample DNA. These points are enlarged upon in the discussion.

**Table 2:** *Phytophthora* species assigned to OTUs in each of two control mixes and the number of reads assigned per OTU by each tool.

*Phytophthora* species assigned per OTU using Swarm	Number of reads/OTU using Swarm	*Phytophthora* species assigned per OTU using Bowtie	Number of reads/OTU using Bowtie
Control mix 1 (15 *Phytophthora* species)			
*P. pseudosyringae/P. ilicis/P. pluvialis/P. nemorosa*	1,595	*P. pseudosyringae*	135
*P. austrocedri*	1,373	*P. austrocedri*	231
*P. kernoviae*	1,311	*P. kernoviae*	216
*P. cactorum/P. idaei*	923	*P. cactorum*	258
		*P. idaei*	1
*P. gonapodyides*	403		
*P. obscura*	383	*P. obscura*	67
*P. ramorum*	173	*P. ramorum*	19
*P. chlamydospora*	116	*P. chlamydospora*	15
*P. lateralis*	100	*P. lateralis*	18
*P. cinnamomi*	80	*P. cinnamomi*	10
*P. cambivora*	48	*P. cambivora*	2
*P. syringae*	5		
*P. mississippiae*	4		
*P. plurivora*	2	*P. plurivora*	2
Control mix 2 (10 *Phytophthora* species)			
*P. capsici/P. glovera/P. Mexicana/P. amaranthi*	1,075	*P. capsici/P. glovera*	147
*P. obscura*	960	*P. obscura*	192
*P. castanae/P. agathacidica/P. cocois/P. sp. novaeguineae*	563	*P. castanae/P. agathacidica/P. sp. novaeguineae*	116
*P. siskiyouensis*	369		
*P. plurivora/P. citricola/P. pachypleura*	342	*P. plurivora*	48
		*P. citricola*	1
*P. foliorum*	320	*P. foliorum*	63
*P. rubi/P. fragariae*	243	*P. rubi*	58
		*P. fragariae*	2
*P. fallax*	216	*P. fallax*	22
*P. cactorum/P. idaei*	33	*P. idaei*	10

Using Swarm as the clustering tool, two of the species included in the first control reaction, *P. pseudosyringae* and *P. ilicis*, could not be distinguished but rather clustered together into a single OTU with 1,595 reads (Table 2). This was due to high sequence similarity as the ITS1 database sequences for these two species differ by only two single nucleotide deletions in *P. ilicis*; one is a deleted A in a run of As and one a deleted C. Two other closely related species with highly similar ITS1 sequences, *P. pluvialis* and *P. nemorosa*, were also falsely assigned to the *P. pseudosyringae* and *P. ilicis* OTU (Table 2). With the exception of *P. boehmeriae*, OTUs were assigned to all remaining *Phytophthora* species included in the first control reaction with the number of reads generated per OTU varying from 1,373 to two (Table 2). A false positive, *P. idaei*, which has a highly similar ITS1 sequence to *P. cactorum*, was assigned to the *P. cactorum* OTU, and an OTU containing four reads was also assigned to a false positive species, *P. mississippiae* (Table 2). These results are likely due to ITS1 sequence variation within the isolates of *P. cactorum* and *P. gonapodyides* included in the control mix, as discussed later. In the second control reaction, *P. boehmeriae* reads were not detected but all remaining species included in the control mix were assigned to OTUs, in some cases together with falsely assigned species of high ITS1 sequence similarity, with read numbers per OTU varying from 1,075 to 33 (Table 2).

Since *P. boehmeriae* sequences were not detected in either control reaction, PCR and Sanger sequencing was used to obtain the ITS1 sequence from the *P. boehmeriae* isolate included in the control mixes. The resulting sequence was identical to the *P. boehmeriae* sequence in the reference database, thus ruling out a sequence mismatch as being the cause for the failure to assign sequences to *P. boehmeriae*. Assembled sequences which had not clustered to the database using Swarm (approximately 8% of the total number of assembled sequences in each control reaction) were compared to sequences in GenBank using BLASTN+ (Altschul et al., 1990), but none matched any sequences of *P. boehmeriae*. Subsequent Illumina sequencing and analysis of reads from a pure culture of *P. boehmeriae* resulted in high numbers of reads being matched to the *P. boehmeriae* sequence in the reference database using the Swarm tool (D.E.L. Cooke, L. Pritchard, P. Thorpe, E. Randall and B. Clark, 2018, unpublished data) indicating the species was detectable using the pipeline. However, there are 3 and 8 bp differences, respectively, between the *P. boehmeriae* ITS sequence and the second round PCR primer sequences, ITS6 F and 5.8S-1R (Scibetta et al., 2012). Inefficient primer annealing may therefore have limited the amplification of the *P. boehmeriae* ITS1 region when included as a component of these species mixes.

The outputs obtained using Swarm as the clustering tool (with a threshold set at 1–2 mismatches across the sequence) allowed for the identification of a greater number of species included in each control reaction compared with the outputs generated using the read-mapping tool Bowtie, which was less sensitive but had fewer false positives (Tables 1 and 2). For example, in some cases where the Swarm output assigned multiple species to an OTU, the Bowtie output assigned a single species to the corresponding OTU (e.g. *P. pseudosyringae* and *P. cactorum/P. idaei* in the first control mix and *P. plurivora* and *P. rubi* in the second control mix) (Table 2). Therefore, in attempting to capture the full range of diversity in the analysis of sequences from the soil samples, the species data reported in this study are those generated using Swarm as the clustering tool, excluding all singleton sequences. The output generated using the Bowtie tool was taken into consideration when multiple closely related species were assigned to an individual OTU using Swarm. If the Bowtie output from the same sample assigned the reads to just one of these species then only this species was included in the data.

### Analysis of sequences from soil samples

Across all 140 soil samples analysed from 14 sites, 112 samples and all sites yielded an amplification product in the nested PCR, with sequences assigned to 35 distinct OTUs in total (Table 3). Trees/shrubs representing 34 plant genera yielded *Phytophthora*-positive soils from around their bases. Of these, approximately 25% were outwardly healthy, 40% showed foliage symptoms typical of *Phytophthora* infection and 35% were either dead or were stumps of hosts felled prior to sampling due to previously confirmed *Phytophthora* infection.

**Table 3:** Distinct *Phytophthora* Operational Taxonomic Units (OTU) found in this study, and species and clade match ranked by number of reads across 14 sites in Scotland.

	Species	Clade	Number of sites	Total	Number of reads per sampled site													
					1	2	3	4	5	6	7	8	9	10	11	12	13	14
1	P. pseudosyringae*/*P. nemorosa/P. pluvialis/P. ilicis*	3	12	154,669	18,563	23,920	136	8,780	6,497	4,022	16,280	–	8,319	–	23,127	20,659	17,944	6,422
2	P. austrocedri*	8	13	74,098	1,490	5,207	6,586	8,584	521	13,857	9,499	2,241	12	–	7,878	16,131	1,525	567
3	P. gonapodyides*	6	11	64,237	9,503	6,584	–	12,732	885	686	5,534	–	1,489	–	7,857	8,542	2,697	7,728
4	P. cambivora*	7	11	40,374	1,277	1,682	–	1,569	6,574	12,949	2,787	–	2,449	–	796	3,006	1,179	6,106
5	P. syringae*	8	10	27,290	785	–	8,600	2,614	4,351	2,669	39	–	3,261	821	–	354	–	3,795
6	*P. ramorum*	8	11	16,491	748	504	–	–	572	1,710	631	4,135	3,136	–	717	894	2,396	1,148
7	P. cactorum*	1	12	15,704	830	891	1,761	3,989	2,743	–	921	–	149	427	1,305	588	895	1,205
8	P. cryptogea*	8	3	8,311	–	–	–	–	–	2	–	7,948	–	–	–	361	–	–
9	*P. chlamydospora*	6	5	8,042	–	552	–	–	–	2,811	208	–	2,911	–	–	426	–	–
10	Unknown sp. 4		6	7,363	1,128	–	–	–	1,571	–	758	–	–	–	–	318	4,550	166
11	Unknown sp. 2	4	2	7,123	5,101	–	–	–	–	–	–	–	–	2,022	–	–	–	–
12	P. plurivora*	2	8	5,845	679	–	–	–	–	2	2	1,878	3,013	203	31	37	–	–
13	P. psychrophila*	3	1	4,761	–	–	–	–	–	–	–	4,761	–	–	–	–	–	–
14	*P. cinnamomi*	7	4	2,593	–	–	–	–	–	650	2	–	719	–	–	1,222	–	–
15	P. uliginosa*/P. europaea*/*P. flexuosa*	7	1	2,545	–	–	–	–	–	–	–	–	–	–	–	–	–	2,545
16	P. primulae*	8	2	2,328	–	–	–	–	–	–	–	–	2,326	–	–	2	–	–
17	Unknown sp. 3	4	1	2,248	–	–	2,248	–	–	–	–	–	–	–	–	–	–	–
18	Unknown sp. 5		1	1,637	–	–	–	–	–	–	–	1,637	–	–	–	–	–	–
19	Unknown sp. 8		1	1,592	–	–	–	1,592	–	–	–	–	–	–	–	–	–	–
20	P. obscura*	8	3	1,273	–	–	–	–	–	–	39	–	–	774	–	–	–	460
21	Unknown sp. 6		2	874	–		–	–	–	–	–	738	–	–	136	–	–	–
22	P. vulcanica*	7	1	830	830	–	–	–	–	–	–	–	–	–	–	–	–	–
23	*P. lateralis*	8	3	713	–	–	–	294	–	197	–	–	222	–	–	–	–	–
24	P. multivora*	2	1	700	–	–	–	–	–	–	–	–	–	–	–	700	–	–
25	Unknown sp. 12		1	666	–	–	–	–	–	–	–	–	–	–	–	–	–	666
26	P. hedraiandra*	1	1	632	–	–	632	–	–	–	–	–	–	–	–	–	–	–
27	Unknown sp. 10	1	1	540	–	–	–	–	–	–	–	–	–	–	–	–	540	–
28	Unknown sp. 9		1	292	–	–	–	–	–	–	–	–	–	–	–	–	292	–
29	Unknown sp. 11		1	249	–	–	–	–	–	–	–	–	–	–	–	–	–	249
30	P. kernoviae*	10	2	226	81	–	145	–	–	–	–	–	–	–	–	–	–	–
31	P. pini*	2	3	100	–	–	–	–	–	–	86	–	–	–	2	12	–	–
32	Unknown sp. 7		1	86	–	–	–	–	–	–	–	–	–	–	–	86	–	–
33	Unknown sp. 1		1	75	75	–	–	–	–	–	–	–	–	–	–	–	–	–
34	P. hibernalis*	8	2	65	–	–	29	–	–	–	–	–	–	–	–	36	–	–
35	P. megasperma*	6	1	50	–	–	50	–	–	–	–	–	–	–	–	–	–	–

**Note:**

* Species known to produce oospores as survival structures.

**Figure 1:**
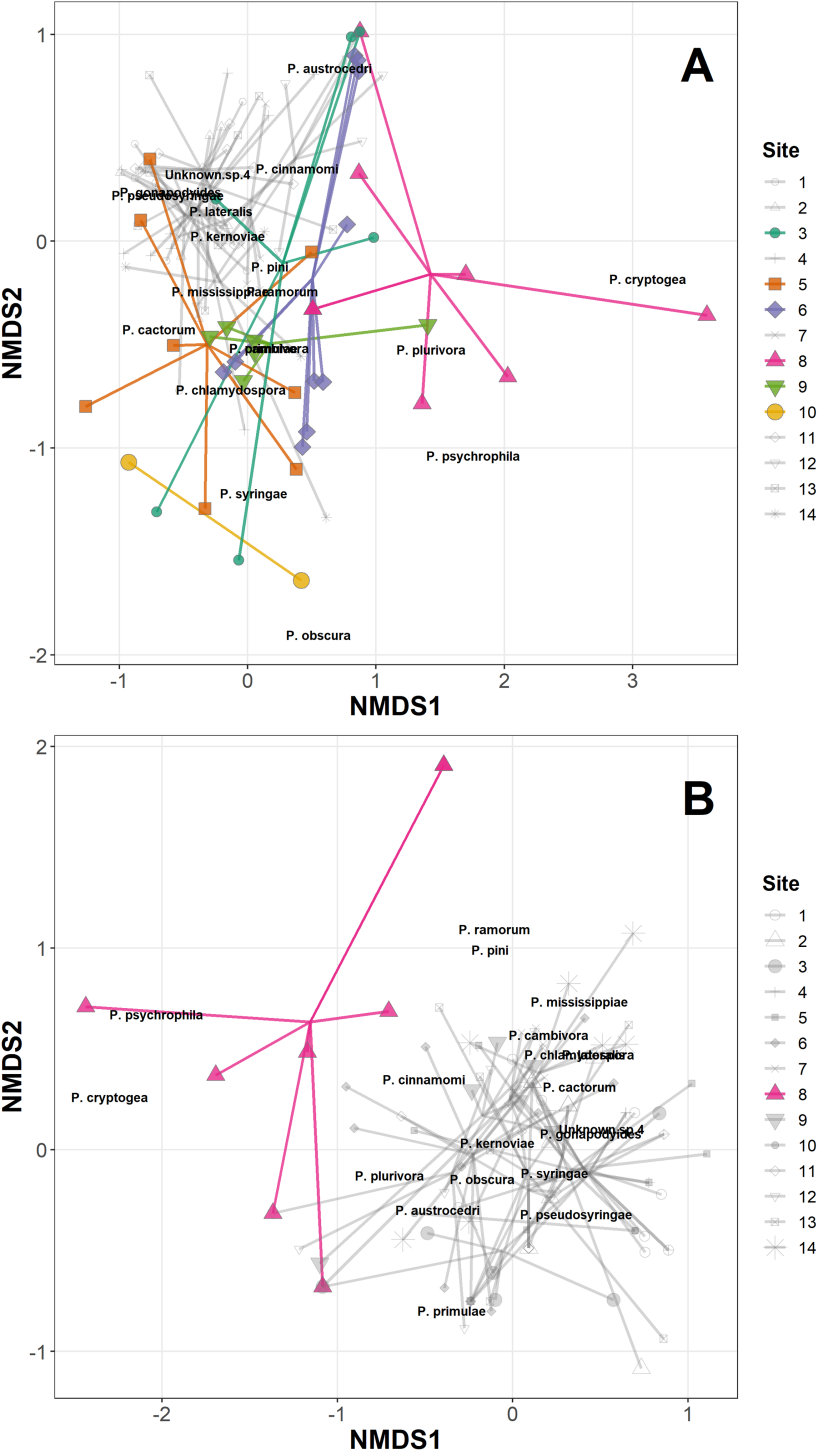
Non-metric multidimensional scaling (NMDS) of *Phytophthora* species abundance data (A) and *Phytophthora* species presence/absence data (B) (Bray–Curtis dissimilarity matrix, with centring and Wisconsin square-root transformations, two of three axes), with ‘ordispider’ plots showing the significant effect of site on the distances (permANOVA, (*F*13,98 = 2.93, *p* < 0.001, abundance data; *F*13,98 = 2.92, *p* < 0.001, presence/absence data)). Those sites occupying markedly different ordination spaces are highlighted (colour, opaque); cluster of sites with similar ordinations are muted (greyscale, transparent).

The permANOVA analysis showed that site-level factors and variables (latitude, longitude, altitude and underlying soil type) were not significant drivers of the variance in the distance matrices in either the *Phytophthora* DNA abundance or absence/presence data (Table S3). Host taxonomic group and health status were not significant drivers within-site, and the only significant factor was site itself, which was highly significant for both the abundance data (*F*_13,98_ = 2.93, *p* < 0.001) and the presence/absence data (*F*_13,98_ = 2.92, *p* < 0.001); Fig. 1A plots the site data onto the first two axes of the NMDS for the abundance data, highlighting in colour sites 1, 5, 6, 8, 9 and 10 which have markedly different ordinations compared with the rest of the sites. Figure 1B presents the NMDS plot for the *Phytophthora* species presence/absence data, highlighting in colour site 8 as markedly different compared with other sites. All sites highlighted in the NMDS plots were found to have within their assemblages either unique species (not found elsewhere in this study) or a high abundance of species consistently found in lesser abundance at other sites. Site 8 in particular yielded DNA of species such as *P. psychrophila*, *P. cryptogea* and two unknown species not found in abundance elsewhere (Figs. 1A and 1B; Table 3).

Generalised linear mixed-effects models also showed no improvement in model fit with the inclusion of site-level variables (latitude, longitude, altitude and underlying soil type), host health status (healthy vs stump/dead/symptomatic) or host taxonomic group on the proportion of samples with DNA of *Phytophthora* species present (applied to those species found in >10 samples in the study) (Table S3). There were significant differences among *Phytophthora* species in terms of overall DNA presence/absence within a sample (Wald χ^2^ = 129, df = 7, *N* = 140, *p* < 0.0001, see Table 4 for post hoc test results).

**Table 4:** Post hoc test results for probability of *Phytophthora* species presence in each sample, calculated from the generalised linear mixed-effects model (binomial errors, probit link function). Values for the Upper CI accompanied by a different letter are significantly different using Tukey’s HSD test.

*Phytophthora* species	P (Presence)	SE	Lower CI	Upper CI
*P. chlamydospora*	0.04	0.02	0.01	0.15^a^
*P. syringae*	0.16	0.06	0.05	0.36^b^
*P. ramorum*	0.17	0.06	0.06	0.38^b^
*P. cactorum*	0.29	0.08	0.12	0.53^b,c^
*P. cambivora*	0.30	0.08	0.13	0.54^b,c^
*P. gonapodyides*	0.40	0.09	0.19	0.64^c,d^
*P. austrocedri*	0.58	0.09	0.34	0.79^d,e^
*P. pseudosyringae/P. P. nemorosa/P. pluvialis/P. ilicis*	0.62	0.09	0.37	0.82^e^

Across all sites and samples, DNA of *P. pseudosyringae/P. nemorosa/P. pluvialis/P. ilicis* and *P. austrocedri* was reported significantly more often in the samples than the six remaining species included in the analysis, except for *P. gonapodyides* which occurred at a similar frequency to *P. austrocedri* (Table 4). Given that neither *P. pluvialis* or *P. nemorosa* are known to occur in the UK (Hansen et al., 2003; Dick et al., 2014; Brar et al., 2017) and *P. ilicis* is only known to infect species within the genus *Ilex* (Buddenhagen & Young, 1957), whereas *P. pseudosyringae* has been reported previously at 23 separate locations in England, Wales and Scotland (Scanu & Webber, 2016), including one of the sites sampled in this study, we consider that the reads assigned by Swarm to *P. pseudosyringae/P. pluvialis/P. nemorosa/P. ilicis* most likely represent *P. pseudosyringae*. This assumption is additionally supported by the assignment of the majority of these sequences to *P. pseudosyringae* by the read-mapping programme Bowtie, for which metapy assigns reads to species only when the alignment has no mismatches.

**Figure 2:**
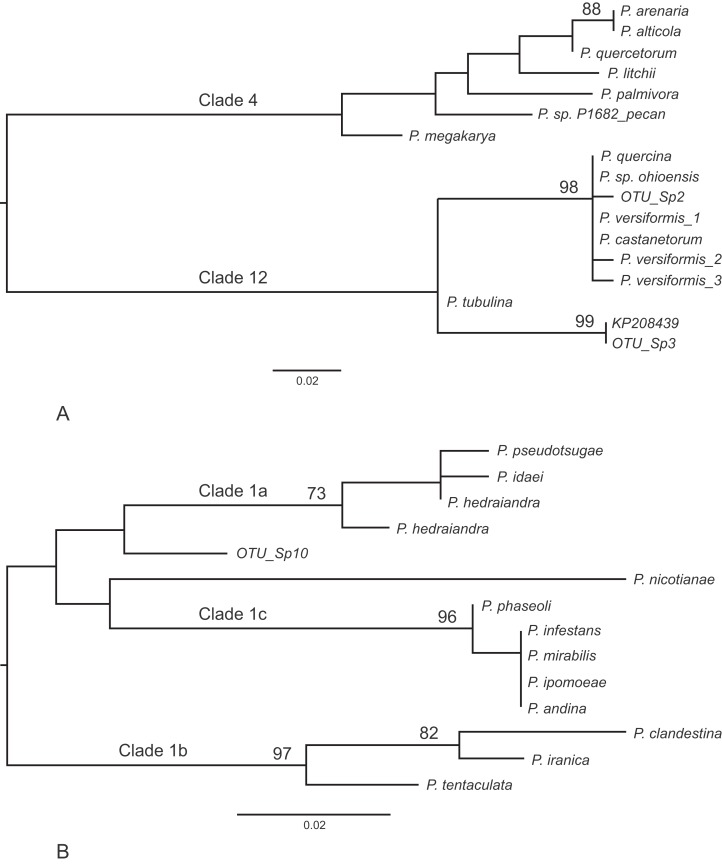
Phylogenetic trees for the ITS1 locus. (A) Position of unknown sp.2 and sp.3 within *Phytophthora* clade 12. Separation of the two clades had 100% bootstrap support. (B) Position of unknown sp.10 within *Phytophthora* clade 1. For both trees bootstrap values higher than 70% are shown. In addition, the internal branch separating clades 4 and 12 was supported by 100% of bootstraps.

Other frequently occurring species identified in the study were *P. cambivora*, *P. cactorum*, *P. ramorum*, *P. syringae* and *P. chlamydospora* (Table 4). Of the other species identified in the study, three also represent *Phytophthora* spp.that are quarantine regulated in the UK (*P. ramorum*, *P. lateralis*, *P. kernoviae*), four are *Phytophthora* spp.not yet reported in the UK (*P. psychrophila*, *P. pini*, *P. vulcanica* and *P. uliginosa/P. europaea/P. flexuosa*) and twelve are oomycete sequences with no match to any known species in the custom curated database or in GenBank nt (Table 5). Phylogenetic analyses placed two of these unknown sequences (sp.2 and sp.3) into the recently designated *Phytophthora* clade 12 (Jung et al., 2017a) (Fig. 2A) and another (sp.10) into *Phytophthora* clade 1 (Fig. 2B). One of the clade 12 sequences (sp.2) differs from sequences of *P. versiformis* and *P. castanetorum* by 1 bp, and from *P. quercina* and *P.sp. ohioensis* by 1 bp and a deletion (Table 5) whereas the other clade 12 sequence (sp.3) differs by 1 bp to a sequence (GenBank accession number KP208439) from an unidentified *Phytophthora* obtained from stream water in the Irati Forest in northern Spain (Català, Pérez-Sierra & Abad-Campos, 2015). The clade 1 sequence (sp.10) differs from any other sequence by at least seven nucleotide differences and two deletions (of 2 and 3 bp). None of the other unknown sequences could be placed within any known *Phytophthora* clade. Two of these sequences (sp.1 and 6) are highly similar to ‘uncultured *Phytophthora*’ sequences arising from metabarcoding studies carried out in forests in Spain (Català, Pérez-Sierra & Abad-Campos, 2015; Català et al., 2017). In terms of sequence identity, one sequence (sp.5) is most similar to *Phytophthora boehmeriae* but differs by 9% (Table 5) and one sequence (sp.7) is most similar to an ‘uncultured oomycete clone’ sequence detected in a metabarcoding study but differs by 11% (Table 5). Three of the sequences (sp.4, 9 and 11) are most similar to the downy mildew species *Plasmopara halstedii* but differ by 9–11% (Table 4) and two of the sequences (sp.8 and 12) are most similar to known sequences in the genus *Pythium*, although in all cases the sequences differ by at least 9% (Table 5).

**Table 5:** Novel OTUs generated in this study; sequence length and closest matches in GenBank.

OTU	Sequence length (bp)	Closest match in GenBank	Match	Reference
Sp.1	178	KP691408: Uncultured *Phytophthora* clone sp4	100% over 178 bp	Català et al. (2017)
Sp.2	184	MF036185: *Phytophthora castanetorum*	99% over 184 bp	Jung et al. (2017a)
		KX011271: *Phytophthora versiformis*	99% over 184 bp	Paap et al. (2017b)
		MH178340: *Phytophthora quercina*	99% over 184 bp	Unpublished sequence: J. Legeay, INRA, France
		HQ261710: *Phytophthora* sp. *ohioensis* voucher P16050	99% over 184 bp	Robideau et al. (2011)
Sp.3	184	KP208439: Uncultured *Phytophthora* clone R2 MOTU14	99% over 184 bp	Català, Pérez-Sierra & Abad-Campos (2015)
Sp.4	283	MF446671: *Phytophthora ramorum* isolate Soil 0010	92% over 224 bp (sites 1–224) and 92% over 127 bp (sites 159-283)	Unpublished sequence: M. van Agtmaal, Imperial College UK
		MF446670: *Plasmopara halstedii* isolate Soil 0007	91% over 224 bp (sites 1–224) and 94% over 123 bp (sites 164-283)	Unpublished sequence: M. van Agtmaal, Imperial College UK
Sp.5	199	KU518801: *Phytophthora boehmeriae* strain OCPC73	91% over 51 bp (sites 1–51)	Unpublished sequence: Madhura et al. Indian Institute of Horticultural Research, India
Sp.6	186	KP208464: Uncultured *Phytophthora* clone R2 MOTU7 from forest soil/water in northern Spain	99% over 186 bp	Català, Pérez-Sierra & Abad-Campos (2015)
Sp.7	172	KY822497: Uncultured oomycete clone OTU201	89% over 54 bp (sites 1–54)	Sapkota & Nicolaisen (2018)
Sp.8	262	KY822489: Uncultured oomycete clone OTU191	90% over 262 bp	Sapkota & Nicolaisen (2018)
		AY598696: *Pythium rostratum* strain CBS 53374	88% over 262 bp	Robideau et al. (2011)
Sp.9	288	MF446670: *Plasmopara halstedii* isolate Soil 0007	89% over 226 bp (sites 1–226) and 96% over 125 bp (sites 165-288)	Unpublished sequence: M. van Agtmaal, Imperial College UK
Sp.10	179	KX423738: *P. cactorum* voucher Chen 32	94% over 179 bp	Chen et al. (2017)
Sp.11	279	MF446670: *Plasmopara halstedii* isolate Soil 0007	90% over 226 bp (sites 1–226)	Unpublished sequence: M. van Agtmaal, Imperial College UK
Sp.12	259	KY822486: Uncultured oomycete clone OTU186	89% over 259 bp	Sapkota & Nicolaisen (2018)
		DQ648134: *Pythium rostratum* isolate P15645	88% over 259 bp	Unpublished sequence: Moralejo et al., School of Engineering of Lullier, Switzerland

Of the 112 soil samples that yielded *Phytophthora* DNA, the mean number of species detected per sample was 4.4 (SD = 2.1), with a maximum of eleven and minimum of one species detected per sample. In terms of clade associations, sequences were assigned to *Phytophthora* species in eight clades; only clades 5 and 9 were not represented (Table 3). Clade 8 was the most represented clade in this study with eight species detected including three well known pathogens of woody hosts (*P. ramorum*, *P. austrocedri*, *P. lateralis*). Of the 23 previously described *Phytophthora* species detected in this study, 19 produce oospores as the main survival structure and four (*P. ramorum*, *P. chlamydospora*, *P. cinnamomi* and *P. lateralis*) produce chlamydospores (Table 3).

### Baiting

Five of the six soil samples collected from site 12 and stored at 4 °C for 7 days prior to baiting yielded *Phytophthora* and the following species were isolated; *P. austrocedri* (UK lineage) from two samples, *P. ramorum* from one sample, *P. cinnamomi* from three samples and *P. multivora* from one sample (Table 6). Following air-drying and 5 weeks storage at 4 °C, three of the six soil samples yielded *Phytophthora* from baits. These were *P. cinnamomi* from three samples and *P. cryptogea* and *P. cambivora*, each from one sample (Table 6). All six *Phytophthora* species found by baiting were among the twelve species detected using metabarcoding 20 months earlier (Table 3; site 12).

**Table 6:** *Phytophthora* species baited from six soil samples collected from a single public garden site in June 2016.

	*Phytophthora* species isolated from six soil samples					
	1	2	3	4	5	6
Soil stored at 4 °C for 1 wk	*P. cinnamomi*	None	*P. ramorum*	*P. multivora*	*P. austrocedri*	*P. austrocedri*
					*P. cinnamomi*	*P. cinnamomi*
Soil stored at 4 °C for 5 wks	*P. cinnamomi*	*P. cinnamomi*	None	None	None	*P. cinnamomi*
	*P. cryptogea*					*P. cambivora*

## Discussion

As far as we are aware this is the first reported study to use Illumina sequencing technology in a metabarcoding approach to assess *Phytophthora* diversity in soil samples. A total of 37 distinct sequences were detected across the 14 sites sampled, corresponding to 23 known *Phytophthora* species including 20 of the 42 species recently listed as present in the UK (Cooke, 2015) as well as four species or species complexes not previously reported in this country. This presents a baseline of expected diversity at highly disturbed sites in Britain.

### Methodology

We included two control reactions to test the consistency of PCR amplification of DNA present and found considerable variability in the number of Illumina reads generated per species. However, this is not unexpected given that the Ct values of the DNA dilutions included in the reactions were not consistent across each species in the mix and that *P. pseudosyringae* and *P. ilicis* sequences were clustered by Swarm into a single OTU in the first control run due to their ITS1 sequence similarity. Although ITS1 sequence length variation across *Phytophthora* species may theoretically affect amplification bias, a previous study carried out to determine whether ITS1 sequence length is inversely related to amplification efficiency of ten *Phytophthora* species showed no relationship between the two (D.E.L. Cooke, 2014, unpublished). DNA from an isolate of *P. boehmeriae* was included in both control mixes but *P. boehmeriae* sequences were not detected. *Phytophthora boehmeriae* is a clade 10 species with an ITS1 sequence that differs markedly (by 15% and 16%, respectively, across 192 bp) from its closest known relatives with ITS sequence data, *P. kernoviae* and *P. morindae*. Due to the presence of several base mismatches in the second round primer regions, we suggest that PCR bias, which can cause false-negative results when sequence variation at the universal primer sites prevents efficient annealing (Pawluczyk et al., 2015), was probably the most likely reason for lack of amplification of *P. boehmeriae* sequences.

In the first control reaction, which included *P. gonapodyides* in the species mix, four sequence reads were assigned using the Swarm tool to an OTU matching *P. mississippiae*. *Phytophthora mississippiae*, which has not previously been recorded in the UK, was recently described from irrigation reservoirs at a plant nursery in Mississippi and is a clade 6 species with a similar ITS sequence to *P. gonapodyides* (Yang, Copes & Hong, 2013). OTUs were also assigned to *P. mississippiae* in two soil samples in this study, both of which yielded a much higher number of sequence reads matching *P. gonapodyides*. Within eukaryotic genomes, rRNA (and hence the ITS1 locus) exists as multiple copies prone to sequence variation with ∼400 copies found in the *P. infestans* genome (Judelson & Randall, 1998). The sequencing depth afforded by Illumina technology would have allowed reads of ITS1 sequences present in low-copy number to be generated, hence it is likely that the so-called ‘*P. mississipiae*’ sequences are in fact being amplified from *P. gonapodyides* and represent a sequence variant present in low-copy number within the genome of the latter species. Similarly, sequence reads matching *P. idaei* and *P. rubi* were likely amplified from *P. cactorum* in the first control mix, and *P. fragariae* in the second control mix, respectively. It is however a possibility that the misidentifications could have occurred due to errors in PCR as the error rate of Taq polymerase is one in three million. Errors might also occur during Illumina sequencing which has an error rate of one in 1,000 bases (Schirmer et al., 2016).

Another false positive reported in the control reaction using Bowtie was *P. citricola* whereas an isolate of the closely related species *P. plurivora* was included in the control mix. *P. plurivora* was described in a taxonomic reclassification of *P. citricola* and only two bp across the ITS1 discriminate the two taxa (Jung & Burgess, 2009). Minor sequence variation or database errors may result in misclassification and some sequences of *P. plurivora* uploaded to GenBank before 2009 are inevitably misidentified as *P. citricola*.

Five species (*P. cactorum*, *P. gonapodyides*, *P. ilicis*, *P. siskiyouensis* and *P. syringae*) included in the control mixes were not identified using the Bowtie tool that requires an exact sequence match to the reference sequence. All five have variable ITS1 sequences reported in GenBank, but only one or two sequences per species were included in the version (June 2017) of the reference database used here. This is an important element for discussion not yet raised in the *Phytophthora* metabarcoding literature. Although the ITS region has proven valuable in *Phytophthora* diagnostics and can be used to distinguish the majority of known species, intraspecific and intra-genomic sequence variation is an important consideration for accurate identification. The reference database should include sequences representative of the known variation within species so that the assignment of sample sequences to OTUs, and species, can be based on appropriate similarity thresholds such as the one to two mismatch threshold used by Swarm here. Bowtie was not the preferred tool for the purpose of this study as it requires a perfect read match and does not allow for the fact that ITS1 sequences of species present in environmental samples might not perfectly match the reference database sequences. However, Bowtie was nonetheless useful for elucidating variation in ITS1 sequence reads. Also, where sequences were assigned using Swarm to multiple closely related species, the output from Bowtie could be informative if it had assigned the same sequences to only one species.

Our control results emphasise the need for further validation and refinement of the reference database and the metapy pipeline. Illumina sequencing of the ITS1 region amplified from pure DNA of known species should be used to identify intraspecific ITS1 sequence variants for subsequent inclusion in the reference database. Such information could also be used to test the efficiency of amplification and clustering of different sequence types. Given that the ITS1 region cannot be used to differentiate a number of very closely related *Phytophthora* species, as reported in this and previous studies (i.e. Català, Pérez-Sierra & Abad-Campos, 2015; Burgess et al., 2017), it would be useful to explore alternative barcoding regions for *Phytophthora*, such as the *cox* mitochondrial genes or a single copy nuclear gene that is conserved within species and yet exhibits sufficient interspecific variation to be able to discriminate species. Current advances in long read sequencing technology such as Pacific Biosciences (PacBio) or Oxford Nanopore technology also offer the prospect of increasing the diagnostic potential of new longer gene regions. For example Redondo et al. (2018b) used PacBio sequencing in a metabarcoding study of *Phytophthora* communities along climatic gradients in Sweden and achieved a high resolution for species identification as a result of being able to sequence both the ITS1 and ITS2 regions. However, PacBio generates fewer sequence reads than Illumina and tends to have higher error rates (Tedersoo, Tooming-Klunderud & Anslan, 2018).

### *Phytophthora* species detected

Taking into account the observed limitations of using ITS1 as the barcode region, we nonetheless consider our approach to have been successful in that the DNA of 23 distinct *Phytophthora* species was detected in soil samples across the 14 sites sampled. Overall, the diversity of species detected was greater than predicted with some species far more widespread than previously thought. As expected, given the nature and *Phytophthora* histories of the sampling locations, all *Phytophthora* species detected, with the exception of *P. primulae*, have documented associations with woody hosts and/or ornamental shrub species.

The geographical location of each site and underlying soil type had no apparent effect on *Phytophthora* species’ presence and abundance, which suggests that the *Phytophthora* communities at each site are largely comprised of introduced species reflecting the range of out planted hosts. There were, however, differences among sites, with site 8 having the most atypical species assemblage. This site was unusual in that it had in the early 2000s been subjected to a very extensive programme of landscaping and replanting, involving the importation of high volumes of containerised plants and soil from continental Europe. The seven most abundant *Phytophthora* species found in this study were detected at the majority of sites, and in soils associated with a range of tree/shrub genera. All seven species, with the exception of *P. austrocedri*, have broad host ranges and three of these species, *P. pseudosyringae*, *P. austrocedri* and *P. ramorum*, are recently invasive and having a severe impact on commercial forest and natural woodland landscapes in the UK. One surprising outcome of this study was that none of the *Phytophthora* species detected in the soil samples were more associated with diseased than healthy hosts. Given that the majority of soils sampled here had been subject to a high turnover of plants, soil and composts over many years, including understorey plantings of annual or perennial herbaceous species, we can assume that the *Phytophthora* assemblages of any one sample reflect the type and source of material brought in to each site, rather than being directly associated with the host tree/shrub/stump central to each sampling point. These findings support those of Redondo et al. (2018a) who analysed *Phytophthora* diversity along a gradient of human interference in Sweden and concluded that invasive *Phytophthora* spp. share a common introduction pathway *via* out-planting of infected nursery stock in urban environments or other sites of high human interference. Population reservoirs are established at these locations with the more ecologically well adapted species able to spread onwards into less populated areas. Additionally, some of the more abundant species found by Redondo et al. (2018a, 2018b) in Sweden were also found in abundance at our Scottish sites; these included *P. cactorum*, *P. cambivora*, *P. gonapodyides*, *P. pseudosyringae*, *P. ramorum* and *P. syringae*.

*Phytophthora pseudosyringae*, the most abundant species detected in this study, is a caducous homothallic species capable of aerial dispersal and was first described in 2003 associated with Fagaceae and *Alnus* hosts in continental Europe (Jung et al., 2003). It is thought to be native to continental Europe, having a scattered but widespread distribution there (Jung et al., 2016). Earlier findings of this species may have been misidentified due to morphological similarities with *P. syringae* (hence the name ‘*pseudosyringae*’) (Jung et al., 2003). Following its formal description, *P. pseudosyringae* was isolated from coastal forests in California and Oregon, associated with the same hosts as *P. ramorum* (Wickland, Jensen & Rizzo, 2008). In the US the pathogen is genetically almost clonal and is hypothesised to be an introduction from Europe (Linzer et al., 2009). *Phytophthora pseudosyringae* was first reported in Britain in 2009 causing disease on *Nothofagus* spp. (Scanu, Jones & Webber, 2012) and *Vaccinium myrtillus* (Beales et al., 2009), and has subsequently been isolated from *F. sylvatica* (Scanu & Webber, 2016) and from aerial stem cankers on *Larix kaempferi* in Britain, often together with *P. ramorum* (J. Webber and A. Harris, personal communication). More recently, *P. pseudosyringae* was isolated for the first time from basal cankers on *Aesculus hippocastanum* in Sweden (Redonodo et al., 2016). Thus the geographical distribution and potential host range of *P. pseudosyringae* appears to be much broader than initially thought and it clearly represents a threat to numerous tree species in Britain. Although this pathogen was first reported in Britain in 2009, its widespread distribution in the country suggests that it has been here for some time (Scanu & Webber, 2016). Isolates collected in Britain and morphologically identified as *P. pseudosyringae* had ITS sequences that shared 100% identity with the ITS sequence of the type strain isolated in Germany in 1997 (Scanu & Webber, 2016). Analyses of the genetic diversity of British isolates across a range of loci will help to shed light on the question of whether *P. pseudosyringae* is native or introduced to this country.

*Phytophthora austrocedri*, the second most abundant species detected in this study, is causing widespread damage to *Juniperus communis* in northern Britain (Green et al., 2015) with all British isolates collected so far conforming to a single clonal ‘UK’ lineage (Henricot et al., 2017). The UK lineage differs genetically and morphologically from the clonal ARG lineage which has expanded in southern Argentina to cause extensive mortality of *Austrocedrus chilensis* (Vélez et al., 2013). To date the known host range of *P. austrocedri* is limited to species within the Cupressaceae, on which it is frequently intercepted in trade (J. Barbrook, Animal and Plant Health Agency, York, England, personal communication; A. Schlenzig, Science and Advice for Scottish Agriculture, Edinburgh, Scotland, personal communication). In this study, the majority of soil samples yielding *P. austrocedri* DNA barcode sequences and the two soil samples from which *P. austrocedri* was baited were collected from around tree/shrub species not documented as hosts for the pathogen. Additionally, ten of the sites represent first-time detections of *P. austrocedri*. If the DNA originates from live propagules, as suggested by the successful baiting of *P. austrocedri* from one of the sites where this pathogen has not previously been reported, then this suggests either that *P. austrocedri* is well adapted to survive in soil in the absence of infected host material, or it can live in association with a much broader range of plant species than is currently thought. Crone et al. (2013a, 2013b) demonstrated the persistence of resting structures of *P. cinnamomi* in the roots of asymptomatic non-hosts and it is quite possible that other *Phytophthora* species could do the same.

The third most abundant *Phytophthora* found in this study was *P. gonapodyides*, a ubiquitous clade 6 species which flourishes in aquatic habitats and is thought to play a role in breakdown of plant debris (Brasier et al., 2003). This species has been isolated from lesions on diseased trees of various species in Britain (Forest Research Tree Health Diagnostic and Advisory Service) although its role as a pathogen in these cases is unclear. Other abundant *Phytophthora* spp. detected here include: *P. cactorum*, *P. cambivora* and *P. syringae*, all of which are considered common in Britain, causing disease on a wide range of woody and non-woody hosts (Cooke, 2015); *P. chlamydospora*, *P. cryptogea* and *P. plurivora*, also regarded as cosmopolitan with broad host ranges (http://forestphytophthoras.org/) (Cooke, 2015), and *P. ramorum*, a quarantine-regulated pathogen causing serious mortality of *Larix* spp. in Britain and with a host range encompassing many shrubby species (Brasier & Webber, 2010).

Of the remaining *Phytophthora* species detected in this study, four (*P. psychrophila*, *P. pini*, *P. vulcanica* and *P. uliginosa/P. europaea/P. flexuosa*) have not previously been reported in the UK. *Phytophthora psychrophila*, found in highest abundance in this study around a dead *C. lawsoniana* at site 8, was first described in 2002 following its isolation from soil in European oak forests (Jung et al., 2002). It is considered to be a weak pathogen able to cause necrotic lesions when artificially inoculated on to roots of young *Quercus robur* (Jung et al., 2002). A subsequent study by Pérez-Sierra et al. (2013) however, found *P. psychrophila* to be partly responsible for root losses and bark cankers in *Quercus* forests in eastern Spain, suggesting that this species has the potential to be more aggressive than initially thought.

*Phytophthora pini* is established in Europe and North America as a pathogen of plants in seven genera including *Pinus* and *Fagus* as well as ornamentals and vegetables, and it is regarded as an increasing threat to the horticultural industry (Jung & Burgess, 2009; Kong et al., 2009; Hong et al., 2011). This pathogen is very closely related to *P. plurivora* and *P. citricola* and could not be distinguished from the former species based on ITS1 alone by Burgess et al. (2017). The ITS1 sequences of *P. pini* and *P. plurivora* included in the reference database used here originate from voucher specimens (GenBank accession numbers: HQ643310 and HQ643312, respectively) and differ by three nucleotides which was sufficient to differentiate the two sequences. *Phytophthora plurivora* was not detected in any of the soil samples yielding sequence reads of *P. pini*. Therefore we believe that our finding of the *P. pini* DNA barcode could indeed represent the first record of *P. pini* in the UK.

A unique sequence identical to GenBank sequences of *P. vulcanica* (MF036209-213) was amplified from a soil sample collected from around a healthy *Castanea sativa* at a single site in this study. *Phytophthora vulcanica* was described very recently following its isolation from rhizosphere soil of *F. sylvatica* in Sicily (Jung et al., 2017a). It was weakly pathogenic to young seedlings of *F. sylvatica* in soil infestation studies, and at this stage is presumed to be endemic to Europe (Jung et al., 2017a).

An OTU containing 2,545 sequence reads assigned to *P. uliginosa/P. europaea/P. flexuosa* was reported at a single site in a soil sample collected from around a *Betula pendula*. *Phytophthora flexuosa* was described recently from Taiwan (Jung et al., 2017b) and is least likely to be the species present here. BLASTN+ analysis of the sequence against GenBank nt showed an equivalent 99% identity with the same single base difference to ITS1 sequences of both *P. uliginosa* and *P. europaea*. The question as to whether it is most likely to be *P. europaea* or *P. uliginosa* present at the site is an interesting one as both species have been found in rhizosphere soils of *Quercus* spp. in Europe (Jung et al., 2002), with *P. europaea* subsequently also isolated from *Quercus* forests in the USA (Balci et al., 2006). Although *P. europaea* was found to be only weakly pathogenic on *Quercus* spp., *P. uliginosa* is reported to be much more aggressive on these hosts (Jung et al., 2002).

Evidence from DNA barcoding suggests a living organism is present but verification of these new records for Britain should be sought by re-sampling the sites and attempting to obtain living cultures from soil or from host material. Vigilance should also be maintained at these sites for disease symptoms. The same applies to sites where the quarantine-regulated pathogens *P. ramorum*, *P. austrocedri* and *P. kernoviae* have been detected for the first time. Conversely, *P. lateralis*, a regulated pathogen of *Chamaecyparis lawsoniana*, was detected in far fewer soil samples than expected given that many were collected from around *C. lawsoniana* with symptoms of active infection at known *P. lateralis* outbreak sites. In the Pacific North West of the USA where *P. lateralis* is invasive and causing high levels of mortality of *C. lawsoniana* in its native range, the pathogen is rarely recovered from soil even in areas of high infestation (Hansen et al., 2000). It can however be baited from the organic fraction of infested soil, leading the authors to conclude that it persists in soil only on infected root fragments, which may not have been retained in the samples processed in the current study.

Three novel *Phytophthora* sequences were identified in this study. Unknown sp.2, which is closest to *P. castanetorum* and *P. versiformis* in clade 12, was amplified from two soil samples; one collected from around a rhododendron and the other from around a pieris, both located at different sites. The correct species identity of our sp.2 remains unknown at present. Sp.3 also appears to be an unknown clade 12 species found in high read numbers from around a healthy rhododendron and Sp.10, which appears to be a novel clade 1 *Phytophthora*, was found around a healthy *Juniperus communis*. Other sequences did not cluster with any known *Phytophthora* clade and included sequences considered to be of downy mildew or *Pythium* origin. The amplification of these sequences from some of the soil samples indicates that the primers used here do cross-react with other oomycetes although the extent of cross reaction was low compared with the much higher abundance of unambiguous *Phytophthora* sequences reported. It should also be noted that two recent phylogenies of the oomycetes have placed the downy mildew species *Plasmopara halstedii*, which aligns most closely with our unknown sp. 4, sp.9 and sp.11, within *Phytophthora* close to *Phytophthora* clade 1 (McCarthy & Fitzpatrick, 2017; Bourret et al., 2018) with the latter authors proposing a new clade 16 within *Phytophthora* in which *Plasmopara halstedii* sits (Bourret et al., 2018).

Clearly, one shortfall of using metabarcoding alone for *Phytophthora* detection is that living cultures are required for absolute verification of the presence and viability of the organism and, in the case of potential new species, for taxonomic studies and host risk analyses to be undertaken. Baiting to obtain living *Phytophthora* isolates was not initially intended to be part of the current study. However, a subsequent visit to one of the sites 20 months after the initial soil samples were collected afforded the opportunity to try to isolate the *Phytophthora* species that had been detected based on DNA data. Baiting at this site, which had never previously reported any *Phytophthora* diseases, yielded six of the 12 *Phytophthora* species identified at the baited sampling points by metabarcoding. This is not surprising given the time lag between the two sampling periods and the low sensitivity of baiting which has a tendency to produce false-negative results (Vannini et al., 2013). Similarly, Khaliq et al. (2018) isolated only six *Phytophthora* species by baiting soil from urban parks in Perth, Australia compared with 13 species detected in the same soil samples using metabarcoding. There may also be a sampling bias inherent to baiting which can favour dominant, fast growing *Phytophthora* species which easily produce zoospores under the baiting conditions and which have a host preference for the baiting material used (Scibetta et al., 2012). Certainly *P. cinnamomi* dominated the isolates obtained by baiting in this study. Additionally, some *Phytophthora* species are regarded as unculturable and so the ability to detect them must rely on molecular methods (Català et al., 2017).

Most of the *Phytophthora* species detected here using metabarcoding produce oospores and not chlamydospores as their main survival structure. Oospores are thick walled and resilient, enabling long-term survival in soil and other substrates without a living host (Ristaino & Gumpertz, 2000). By only sampling soil, our approach might have been biased towards homothallic species that readily produce oospores, whereas water sampling may yield different or a greater variety of species (Català, Pérez-Sierra & Abad-Campos, 2015; Redondo et al., 2018a). Research is currently underway to compare metabarcoding and baiting approaches for analyses of *Phytophthora* diversity in both soil and water samples from a range of sites across Britain, including adjacent disturbed and less disturbed environments. It is quite likely that future protocols will use a refined combination of methods for screening environmental samples for *Phytophthora* pathogens.

## Conclusions and Future Considerations

This study has demonstrated the ubiquity and diversity of *Phytophthora* species endemic in highly managed, extensively planted soils in northern Britain. If the DNA detected represents the presence of, in most cases, live propagules then this also highlights the versatility of this genus of plant pathogens in terms of survival in soil and potential transfer to susceptible hosts. Many of the *Phytophthora* species found here have previously been recorded in plant nurseries across Europe (Jung et al., 2016), and thus our findings highlight the potential risks posed by spread of *Phytophthora*-contaminated soil and planting material. Our study also supports the recommendations of Paap, Burgess & Wingfield (2017a) and Hulbert et al. (2017) that botanic garden and amenity woodland sites in, or close to, urban areas could be used as sentinel plantings and targeted for early detection of invasive pathogens, particularly where citizen science monitoring programmes could be utilised. The fact that a high diversity of species was frequently detected in each individual soil sample, in two cases eleven distinct species, also raises questions about possible interactions between species co-inhabiting these soil inoculum reservoirs, and the potential risks of new genotypes arising through horizontal gene transfer and hybridisation events. It is evident that managers of public parks and gardens need to establish good hygiene practice to avoid transfer of soil-borne *Phytophthora* spp. within site, and from one site to another. This might involve, for example, keeping public access to hard-core pathways and ensuring staff clean boots, tools and vehicles before moving from one location to another on the site. Steps should also be taken to ensure that healthy planting stock is sourced from reputable nurseries with a documented disease management plan. Our study has also highlighted where potential errors might occur in the bioinformatic analyses and where further refinement of the method is required, emphasising the importance of prior knowledge, both in terms of sample origin and *Phytophthora* species behaviour, in enabling correct and useful biological interpretation of the bioinformatic data. Finally, this study has demonstrated the potential power and utility of metabarcoding as part of surveillance strategies for invasive pathogens, strengthening the evidence base for more effective biosecurity and remediation policy aimed at limiting the introduction, spread and impact of *Phytophthora* diseases both into, and within, regions.

## Supplemental Information

10.7717/peerj.6931/supp-1 Supplemental Information 1 Approximate altitude at centre and underlying soil type for the 14 sites sampled in this study.

10.7717/peerj.6931/supp-2 Supplemental Information 2 GenBank accession number, clade and isolate identification for sequences used in the phylogenetic analyses.

10.7717/peerj.6931/supp-3 Supplemental Information 3 Results of permANOVA and GLiMMs analyses.

## Additional Information and Declarations

### Data Availability

The following information was supplied regarding data availability: The raw data are available in the Tables.
