# Supplementary material for: Metabarcoding reveals a high diversity of woody host-associated Phytophthora spp. in soils at public gardens and amenity woodlands in Britain
Source: PeerJ. 2019 May 16;7:e6931. doi: 10.7717/peerj.6931 (PMC6526010; doi:10.7717/peerj.6931)
Supplement: Supplemental Information 1 [file peerj-07-6931-s001.docx]

Table S1. Approximate altitude at centre and underlying soil type for the fourteen sites sampled in this study, and the number of soil samples collected at each site (n=10) from around trees/shrubs in each of six broad taxonomic groupings and two health status categories.

|  |  |  | Tree/shrub broad taxonomic grouping and health status (H, healthy; D, symptoms/stump/dead) | | | | | | | | | | | |
| --- | --- | --- | --- | --- | --- | --- | --- | --- | --- | --- | --- | --- | --- | --- |
|  |  |  | Cupressaceae | | Other conifers | | Ericaceae | | Fagaceae/  Nothofagaceae | | Other angiosperms | | Other | |
| Site | Altitude (m) | Underlying soil type | H | D | H | D | H | D | H | D | H | D | H | D |
| 1 | 30 | Brown earth, moderately well drained | 0 | 1 | 0 | 1 | 0 | 4 | 2 | 1 | 0 | 1 | 0 | 0 |
| 2 | 55 | Urban | 1 | 7 | 0 | 2 | 0 | 0 | 0 | 0 | 0 | 0 | 0 | 0 |
| 3 | 45 | Brown earth, well drained | 1 | 0 | 0 | 0 | 2 | 4 | 1 | 0 | 0 | 2 | 0 | 0 |
| 4 | 20 | Urban | 0 | 10 | 0 | 0 | 0 | 0 | 0 | 0 | 0 | 0 | 0 | 0 |
| 5 | 100 | Urban | 2 | 6 | 0 | 2 | 0 | 0 | 0 | 0 | 0 | 0 | 0 | 0 |
| 6 | 5 | Poorly drained peat | 1 | 1 | 0 | 1 | 2 | 2 | 0 | 0 | 0 | 1 | 2 | 0 |
| 7 | 105 | Brown earth, humus iron podzols, well drained | 0 | 1 | 0 | 0 | 0 | 0 | 0 | 7 | 0 | 2 | 0 | 0 |
| 8 | 45 | Brown earth, moderately well drained | 2 | 4 | 0 | 4 | 0 | 0 | 0 | 0 | 0 | 0 | 0 | 0 |
| 9 | 15 | Humus iron podzols, well drained | 1 | 1 | 2 | 0 | 1 | 4 | 1 | 0 | 0 | 0 | 0 | 0 |
| 10 | 30 | Humic gleys, poorly drained | 0 | 2 | 2 | 2 | 1 | 1 | 0 | 1 | 0 | 1 | 0 | 0 |
| 11 | 80 | Brown earth, well drained | 0 | 1 | 1 | 0 | 1 | 4 | 0 | 0 | 0 | 3 | 0 | 0 |
| 12 | 30 | Brown earth, moderately well drained | 0 | 2 | 0 | 1 | 0 | 2 | 0 | 0 | 3 | 1 | 1 | 0 |
| 13 | 300 | Peaty gleys, poorly drained | 2 | 8 | 0 | 0 | 0 | 0 | 0 | 0 | 0 | 0 | 0 | 0 |
| 14 | 30 | Urban | 1 | 1 | 1 | 0 | 0 | 2 | 1 | 1 | 0 | 3 | 0 | 0 |
