# Supplementary material for: Metabarcoding reveals a high diversity of woody host-associated Phytophthora spp. in soils at public gardens and amenity woodlands in Britain
Source: PeerJ. 2019 May 16;7:e6931. doi: 10.7717/peerj.6931 (PMC6526010; doi:10.7717/peerj.6931)
Supplement: Supplemental Information 2 [file peerj-07-6931-s002.docx]

| **Accession number** | **Clade** | | **Isolate** |
| --- | --- | --- | --- |
| Figure 2a sequences | |  |  |
| KP208439 | 12 | | Uncultured *Phytophthora* clone R2 MOTU14 |
| AJ131986 | 12 | | *P. quercina* IFB-QUE4 |
| MF170966 | 12 | | *P. versiformis*_1 TP13.49 |
| MF170964 | 12 | | *P. versiformis*_2 TP13.12 |
| MF170963 | 12 | | *P. versiformis*_3 TP13.11 |
| MF036182 | 12 | | *P. castanetorum* BD292 |
| HQ261710 | 12 | | *P. sp. ohioensis* voucher P16050 |
| MF036196 | 12 | | *P. tubulina* TUB1 |
| GU111615 | 4 | | *P. litchii* TARI 28165 |
| EF539174 | 4 | | *P. quercetorum* WV5-6 18S |
| GU997621 | 4 | | *P.* sp.P16825_pecan |
| HQ013219 | 4 | | *P. arenaria* CBS 127950 |
| GU111659 | 4 | | *P. palmivora* TARI 26001 |
| AF266782 | 4 | | *P. megakarya* IMI337104 |
| HQ013214 | 4 | | *P. alticola* CMW34279 |
|  |  | |  |
| Figure 2b sequences | | |  |
| KJ494916 | 1 | | *P. nicotianae* QS-2 |
| AF266772 | 1a | | *P. cactorum* IMI296524 |
| AY707987 | 1a | | *P. hedraiandra* CBS 111725 |
| AF266774 | 1a | | *P. pseudotsugae* IMI331662 |
| AF266773 | 1a | | *P. idaei* IDA3 |
| AJ131989 | 1b | | *P. clandestina* CLA2 (IMI287317) |
| AJ131987 | 1b | | *P. iranica* IRA1 (IMI158964) |
| AF266775 | 1b | | *P. tentaculata* CBS552.96 |
| AY770741 | 1c | | *P. andina* EC3363 |
| MG865514 | 1c | | *P. infestans* CPHST BL 19 |
| MG865518 | 1c | | *P. ipomoeae* Ex-type CPHST BL 21 |
| AF266778 | 1c | | *P. phaseoli* CBS556.88 |
| AF266777 | 1c | | *P. mirabilis* CBS678.85 |

Supplementary Table 2. GenBank accession number, clade and isolate identification for sequences used in the phylogenetic analyses.
