# Supplementary material for: Metabarcoding reveals a high diversity of woody host-associated Phytophthora spp. in soils at public gardens and amenity woodlands in Britain
Source: PeerJ. 2019 May 16;7:e6931. doi: 10.7717/peerj.6931 (PMC6526010; doi:10.7717/peerj.6931)
Supplement: Supplemental Information 3 [file peerj-07-6931-s003.docx]

Table S3. Results of permANOVA and GLiMMs analyses

permANOVA: Abundance data

|  | Df | SumsOfSqs | MeanSqs | F.Model | R2 | Pr(>F) |
| --- | --- | --- | --- | --- | --- | --- |
| scale(Lat) | 1 | 0.8556873 | 0.8556873 | 2.709803 | 0.0231955 | 1 |
| scale(Long) | 1 | 0.4096080 | 0.4096080 | 1.297152 | 0.0111034 | 1 |
| scale(Alt.m.) | 1 | 0.8417449 | 0.8417449 | 2.665650 | 0.0228175 | 1 |
| Drainage | 3 | 1.6268964 | 0.5422988 | 1.717360 | 0.0441009 | 1 |
| Residuals | 105 | 33.1563484 | 0.3157747 | NA | 0.8987827 | NA |
| Total | 111 | 36.8902850 | NA | NA | 1.0000000 | NA |

permANOVA: Site effects: Abundance data

|  | Df | SumOfSqs | F | Pr(>F) |
| --- | --- | --- | --- | --- |
| Host.Status2 | 1 | 0.3085679 | 1.138869 | 0.322 |
| Host.Group | 5 | 1.3605765 | 1.004329 | 0.481 |
| Site | 13 | 9.9604715 | 2.827872 | 0.001 |
| Residual | 92 | 24.9266911 | NA | NA |

permANOVA: Site-level effects: Abundance data

|  | Df | SumsOfSqs | MeanSqs | F.Model | R2 | Pr(>F) |
| --- | --- | --- | --- | --- | --- | --- |
| Site | 13 | 10.32063 | 0.7938947 | 2.928216 | 0.2797655 | 0.001 |
| Residuals | 98 | 26.56965 | 0.2711189 | NA | 0.7202345 | NA |
| Total | 111 | 36.89028 | NA | NA | 1.0000000 | NA |

permANOVA: Presence/absence data

|  | Df | SumsOfSqs | MeanSqs | F.Model | R2 | Pr(>F) |
| --- | --- | --- | --- | --- | --- | --- |
| scale(Lat) | 1 | 0.3911757 | 0.3911757 | 1.610005 | 0.0134113 | 1 |
| scale(Long) | 1 | 0.4365659 | 0.4365659 | 1.796822 | 0.0149675 | 1 |
| scale(Alt.m.) | 1 | 0.8055457 | 0.8055457 | 3.315472 | 0.0276178 | 1 |
| Drainage | 3 | 2.0229644 | 0.6743215 | 2.775378 | 0.0693565 | 1 |
| Residuals | 105 | 25.5113879 | 0.2429656 | NA | 0.8746470 | NA |
| Total | 111 | 29.1676396 | NA | NA | 1.0000000 | NA |

permANOVA: Site-level effects: Presence/absence data

|  | Df | SumOfSqs | F | Pr(>F) |
| --- | --- | --- | --- | --- |
| Host.Status2 | 1 | 0.3250616 | 1.530754 | 0.136 |
| Host.Group | 5 | 1.1336902 | 1.067737 | 0.359 |
| Site | 13 | 7.6738460 | 2.779774 | 0.001 |
| Residual | 92 | 19.5365572 | NA | NA |

permANOVA: Site-level effects: Presence/absence data

|  | Df | SumsOfSqs | MeanSqs | F.Model | R2 | Pr(>F) |
| --- | --- | --- | --- | --- | --- | --- |
| Site | 13 | 8.149061 | 0.6268508 | 2.922718 | 0.279387 | 0.001 |
| Residuals | 98 | 21.018579 | 0.2144753 | NA | 0.720613 | NA |
| Total | 111 | 29.167640 | NA | NA | 1.000000 | NA |

GLiMM: ANOVA style comparison of initial and final model (plus AIC)

|  | Df | AIC | BIC | logLik | deviance | Chisq | Chi Df | Pr(>Chisq) |
| --- | --- | --- | --- | --- | --- | --- | --- | --- |
| Final GLiMM | 10 | 1177.628 | 1227.839 | -578.8139 | 1157.628 | NA | NA | NA |
| Initial GLiMM | 31 | 1190.806 | 1346.460 | -564.4031 | 1128.806 | 28.8215 | 21 | 0.1183534 |

Final GLiMM: ANOVA

|  | Chisq | Df | Pr(>Chisq) |
| --- | --- | --- | --- |
| Phyt | 129.14 | 7 | 0 |
